# Ease of Using a Dedicated Percutaneous Closure Device after Inadvertent Cannulation of the Subclavian Artery: Case Report

**DOI:** 10.1155/2009/728629

**Published:** 2009-06-17

**Authors:** Arnaud Devriendt, Emmanuel Tran-Ngoc, Philippe Gottignies, José Castro-Rodriguez, Oliver Lomas, Sophie Jamart, Sébastien Knecht

**Affiliations:** ^1^Department of Cardiology, CHU Brugmann, 1020 Brussels, Belgium; ^2^Department of Critical Care, CHU Brugmann, 1020 Brussels, Belgium

## Abstract

Inadvertent puncture of the subclavian artery is a relatively frequent and potentially disastrous complication of attempted central venous access. Due to its noncompressible location, accidental subclavian arterial cannulation may result in hemorrhage as the sheath is removed. We report a new case of successful percutaneous closure of the subclavian artery which had been inadvertently cannulated, using a closure device based on a collagen plug (Angio-Seal, St. Jude Medical). This was performed in a patient who had received maximal antiplatelet and anticoagulation therapies because of prior coronary stenting in the context of cardiogenic shock. There was no prior angiographic assessment, as arterial puncture was presumed to have been distal to the right common artery and vertebral arteries. No complications were observed in this high-risk patient, suggesting that this technique could be used once the procedure has been evaluated prospectively.

## 1. Case Report

A 68-year-old woman with a history of arterial hypertension was admitted at the Cardiology Department because of massive anterior myocardial infarction with subsequent cardiogenic shock. After initial manoeuvres including sedation, mechanical ventilation, and catecholamine infusion, she was assessed for urgent coronary angiography. Using intra-aortic balloon pump counterpulsation support, coronary angiography allowed treatment of critical stenosis of the left interventricular and right arteries. The patient was then transferred to the Intensive Care Unit, and received maximal antiplatelet (clopidogrel and aspirin) and anticoagulation (low-molecular-weight heparin) therapy. Of note, arterial accesses at the both right and left femoral groins were maintained.

**Figure 1:**
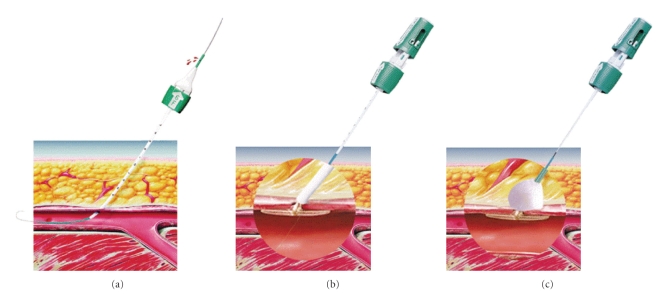
Description of the Angio-Seal device. (a) Introduction of a dedicated wire in the artery, followed by the insertion of a percutaneous closure device with an arteriotomy locator. (b) and (c) The device creates a mechanical seal by sandwiching the arteriotomy between a bioabsorbable anchor and collagen sponge, which dissolve within 60 to 90 days.

An attempt at placing a 7.5F central venous catheter in the right subclavian vein was carried out for monitoring and infusions. This resulted in inadvertent cannulation and insertion of the 7.5F sheath into the right subclavian artery. The poor hemodynamic condition of the patient precluded invasive open surgery, and a decision was made to attempt arterial percutaneous closure, with an 8F collagen plug-based closure device (Angio-Seal, St. Jude Medical) (Figure 1). Angiography of local arteries was not performed because arterial puncture had been made distal to the right common artery and vertebral arteries. 

**Figure 2:**
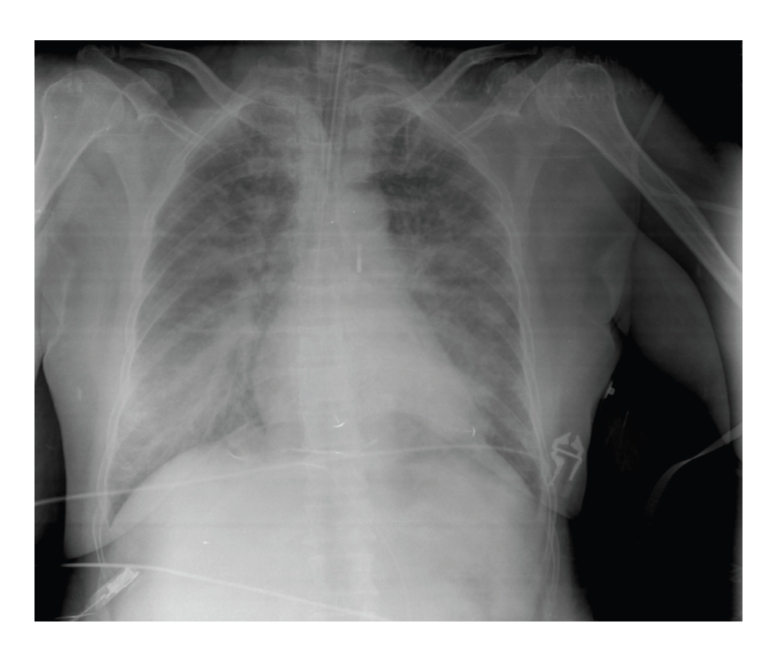
Chest X-ray after Angio-Seal placement.

A dedicated 0.035 J-wire was then introduced through the catheter in the artery, which allowed removal of the sheath and insertion of the percutaneous closure device with an arteriotomy locator. The dilatator was then withdrawn and the Angio-Seal device was subsequently inserted and deployed. The patient showed no sign of local hemorrhage or arterial occlusion. A repeat radiograph of the chest excluded hemorrhagic complications including hemothorax (Figure 2). Antiplatelet and anticoagulation therapies could be maintained to preserve the coronary flow. The situation of the patient continued to improve, allowing for removal of mechanical ventilation after 5 days and catecholamine therapy after 7 days. She was discharged from the Intensive Care Unit 28 days following the deployment of the Angio-Seal positioning.

## 2. Discussion

Inadvertent puncture of the subclavian artery occurs in up to 2.7% of the cases during central venous cathaterization using a subclavian venous approach [1]. Mainly because of its noncompressible location, accidental subclavian arterial cannulation may result in potentially disastrous complications such as hemorrhage, subclavian occlusion, embolism and pseudoaneurysm formation, or local nervous compression secondary to hematoma formation. These risks are majored in critically ill cardiac patients, especially those on systemic anticoagulation and receiving major antiplatelet agents.

Different techniques have been described in the case of subclavian artery cannulation. In addition to surgery and placement of a covered stent, percutaneous closure devices have been reported to be generally safe [2–4], although no prospective trials have already been made in this field. In particular, Sharma et al. [5] described a case where deployemnt of a closure device resulted in an abrupt occlusion of the subclavian artery, necessitating use of a balloon and a throbectomy to restore arterial blood flow. In our case, no prior angiography was performed because the puncture was considered to be located distal to the carotid and vertebral arteries. Of note, arterial access had been maintained and this could be used for percutaneous balloon tamponnade or placement of a covered stent in the case of local hemorrhage. Furthermore, this had been carried out by experienced cardiologists with the support of vascular surgeons, prompt to use alternative technique in case of complication. 

Our case is unusual because this patient was at high risk of hemorrhagic complications due to the antiplatelet and anticoagulation therapies. However, the Angio-Seal device is already widely and safely used to close femoral artery in patients with high dose antiplatelet and anticoagulation therapies after percutaneous coronary stenting [6]. Furthermore, related complications following Angio-Seal positioned in the femoral artery are as rare as manual compression [7, 8]. These include hematoma, local bleeding requiring transfusion, pseudoaneurysm, arteriovenous fistula and retroperitoneal hemorrhage, occlusion of the femoral artery, and plug embolization or infection.

## 3. Conclusion

We report a successful closure of an inadvertent cannulation of the subclavian artery in a patient with high hemorrhagic risk, using a closure device based on a collagen plug. This technique appears to be effective and safe although this prospective evaluation in a large trial is required. Furthermore, this must be carried out by experienced cardiologists, who are ready to use alternative techniques in case of complication with this percutaneous approach.
